# Effects of phorbol myristate acetate and sivelestat on the lung injury caused by fat embolism in isolated lungs

**DOI:** 10.1186/1423-0127-19-3

**Published:** 2012-01-05

**Authors:** Chia-Chih Lin, Pei-Hsin Liu, Shang Jyh Kao, Hsing I Chen

**Affiliations:** 1 Department of Physical Education and Kinesiology, National Dong Hwa University, Hualien, Taiwan; 2 Department of Anatomy, Tzu Chi University, Hualien, Taiwan; 3 School of Respiratory Therapy, Fu Jen Catholic University and Taipei Medical University, New Taipei City, Taiwan; 4 Department of Internal Medicine, Shin Kong Wu Ho-Su Memorial Hospital, New Taipei City, Taiwan; 5 Institute of Physiological and Anatomical Medicine, Tzu Chi University, Hualien, Taiwan

**Keywords:** Fat embolism, Acute lung injury, Neutrophil elastase, Phorbol myristate acetate, Sivelestat

## Abstract

**Background:**

Fat embolism syndrome (FES) associated with acute lung injury (ALI) is a clinical condition following long bone fracture. We have reported 14 victims due to ALI with FES. Our laboratory has developed an animal model that produced fat emboli (FE). The major purpose of this study was to test whether neutrophil activation with phorbol myristate acetate (PMA) and inhibition with sivelestat (SVT) exert protection on the lung.

**Methods:**

The lungs of Sprague-Dawley rats were isolated and perfused. FE was produced by addition of corn oil micelles into the lung perfusate. PMA and SVT were given simultaneously with FE. Parameters such as lung weight/body weight ratio, LW gain, exhaled nitric oxide (NO), protein concentration in bronchoalveolar lavage relating to ALI were measured. The neutrophil elastase (NE), myeloperoxidase, malondialdehyde and phopholipase A_2 _activity were determined. We also measured the nitrate/nitrite, methyl guanidine (MG), and cytokines. Pulmonary arterial pressure and microvascular permeability were assessed. Lung pathology was examined and scored. The inducible and endothelial NO synthase (iNOS and eNOS) were detected.

**Results:**

FE caused ALI and increased biochemical factors. The challenge also resulted in pulmonary hypertension and increased microvascular permeability. The NE appeared to be the first to reach its peak at 1 hr, followed by other factors. Coadministration with PMA exacerbated the FE-induced changes, while SVT attenuated the effects of FE.

**Conclusions:**

The FE-induced lung changes were enhanced by PMA, while SVT had the opposite effect. Sivelestat, a neutrophil inhibitor may be a therapeutic choice for patients with acute respiratory distress syndrome (ARDS) following fat embolism.

## Background

Fat embolism syndrome (FES) is a serious clinical problem in patients associated with long bone fracture [1-3]. Although the precise mechanisms of FES remain unclear, intravasation of fat or fatty acids from broken long bones and other sources is the primary cause leading to FES [3,4]. In two clinical investigations, we have reported a total of 14 cases who died of acute respiratory distress syndrome (ARDS) associated with FES [1,3]. The occurrence of ARDS following FES suggests that the lung is one of the target organs following intravasation of fat emboli [1-4].

In order to elucidate the possible mediators involved in the ARDS associated with FES, we have developed an animal model that produces fat embolism in anesthetized rats. Intravenous administration of corn oil micelles induces alveolar edema and hemorrhage. The pathological changes are associated with fatty droplets and fibrin thrombi in the lung, kidney and brain. The arteriolar lumen is filled with fatty deposits. Hypoxia and hypercapnia ensue. Biochemical changes include increases in plasma phospholipase A_2_, nitrate/nitrite, methyl guanidine and proinflammatory cytokines [5]. The animal model has been used to study the protective effect of N-Acetylcysteine [6], and the effects of inducible nitric oxide synthase (iNOS) inhibitors and nitric oxide donors [7]. Our results indicate that N-acetylcysteine provides protection to the FES, while nitric oxide is detrimental.

Activation and recruitment of neutrophil that lead to the release of neutrophil elastase (NE) and other toxic mediators may play an initial role in the pathogenesis of ALI/ARDS [8-10]. Accumulating evidence has indicated the involvement of neutrophil activation and NE induced by phorbol myristate acetate (PMA) and other agents [11-13]. Animal experimentation has demonstrated that sivelestat (SVT), an inhibitor of NE attenuates ALI via reduction of NE following lipopolysaccharide administration or inhalation [14,15], and cardiopulmonary bypass [16,17].

The present study was designed to test whether neutrophil activation with PMA and inhibition with SVT exert protective and/or detrimental effects on the acute lung injury caused by fat embolism.

## Materials and methods

### Animal preparation

We used male Spague-Dawley (SD) rats, 12-14 wk-old, weighing 360-380 g. The animals were obtained from the National Animal Center and housed in the University Laboratory Animal Center with good environment control. The animal experiment was approved by the University Committee of Laboratory Animal Care and Use, and followed the guidelines of the National Animal Research Center. The room temperature was maintained at 21 ± 1°C under a 12/12 hr light/dark regimen. Food and water were provided *ad libitum*.

### Isolation and perfusion of the lung *in situ*

We followed the procedures for the preparation of isolated and perfused rat's lungs *in situ *[7,18]. In brief, the rat's lungs were isolated and perfused with constant flow. Lung weight (LW) and LW gain (LWG) were recorded. Pulmonary arterial pressure (PAP) and pulmonary venous pressure (PVP) were measured.

### Microvascular permeability (K_fc_)

Capillary filtration coefficient (K_fc_) as an index of microvascular permeability was calculated from the increase in LW produced by an elevation in PVP. The K_fc _was defined as the initial weight gain rate (g/min) divided by PVP (10 cm H_2_O) and LW, and expressed as g/min/cmH_2_O/100 g. During the experiment, PVP was rapidly elevated by 10 cm H_2_O for 7 min to measure K_fc_. This hydrostatic challenge elicited a biphasic increase in LW: an initial rapid component, followed by slow and steady component. The slow component of the weight gain was plotted on a semilog scale as a function of time. The capillary filtration rate was obtained by extrapolating the slow component of the weight gain back to zero time [7,18].

### Exhaled NO

An increase in exhaled NO concentration has been used as an early marker of lung inflammation or injury [19,20]. We measured the NO concentration in expired air. A specimen of exhaled air (300 ml in 30 min) was suctioned into a gas purge chamber previously evacuated to remove oxygen. The NO concentration was rapidly determined after air collection. The measurement of NO with a chemiluminescence analyzer (Sievers 270B NOA; Sievers Institute, Denver, CO, USA) was based on the principle that NO interacts with ozone to generate chemiluminescent light. The chemiluminescence is directly proportional to the NO level. In addition to a photomultiplier tube, an ozone generator and a gas chamber were included. The ozone generator was used to produce ozone internally. The exhaled NO was measured every 30 min after introduction of corn micelles into the lung perfusate. It reached its peak depending on the experimental conditions. The peak value was taken as the NO concentration.

### Protein concentration in bronchoalveolar lavage (PCBAL)

After the experiment, lungs were lavaged twice with saline (2.5 ml per lavage). Lavage samples were centrifuged at 1,500 g at room temperature for 10 min. The PCBAL was determined with a spectrophotometer by measuring the change in absorbance at 630 nm after the addition of bromocresol green [6,20].

### Neutrophil elastase, myeloperoxidase, malondialdehyde and phospholipase A_2 _activity

The neutrophil elastase (NE) in lung perfusate was determined with a synthetic substrate, N-methoxysuccinyl-Ala-Ala-Pro-Val-p-nitroanilide as described previously [6,11]. In brief, samples were incubated in 0.1 M Tris-HCl buffer (pH 8.0) containing 0.5 M NaCl and 1 mM substrate at 37°C for 24 hr. After incubation, p-nitroanilide release was measured spectrophotometrically at 450 nM and was considered NE activity.

To measure the myeloperoxidase (MPO) activity in lung perfusate, the samples were mixed with 2 ml of potassium phosphate buffer (50 mM, pH 6.0) containing 0.5% cetyltrimethylammonium bromide and were centrifuged at 2,500 g for 10 min at 4°C. The supernatant was diluted with dilution buffer, then mixed with an assay buffer composed of 0.00107% H_2_O_2 _in potassium phosphate buffer and o-dianisidine. The reaction mixture was incubated at room temperature. The change in absorbance at 450 nm over 1 min was detected spectrophotometrically. The MPO activity was expressed as units per ml of lung perfusate using the absorbance of MPO standard (Elastine Products, Detroit, MC., USA). The procedures were basically followed those by Kinoshita et al. [8].

Malondialdehyde (MDA) was measured by thiobarbituric acid reaction. The principle of the method depends on the development of pink color produced by the interaction of barbituric acid with MDA as a result of lipid peroxidation. Tetraetoxypropane was used as standard [21,22].

Plasma concentrations of phospholipase A_2 _(PLA_2_) were measured on a spectrofluorimeter using a method described by Kitsiouli et al. [23]. In brief, the standard incubation mixture contained 10 mmol/l tris/HCl buffer (pH 7.4) with 2 mmol/l Ca2^+ ^and 5 *μ*mil/l C_12_-NBD-PC{1-palmitoyl-2-[12-[(7-nitro-2-1,3-benzoxadiazol-4-yl)amino]hexanoyl]-*sn*-glycero-3-phosphocholine} as the substrate. The absorbance of reaction mixture was measured with excitation and emission wavelengths at 475 and 535 nm, respectively.

### Nitrate/nitrite, methyl guanidine, tumor necrosis factor_α _and interleukin-1_β_

Samples (0.5 ml) were taken from the lung perfusate 1 hr before and at various time points after drug administration. The samples were centrifuged at 3,000 g for 10 min. The supernatant was used for determination of nitrate/nitrite with high-performance chromatography [20,24]. The formation of methyl guanidine (MG) has been identified as an index of hydroxyl radical production [6,25]. It was determined with its fluorescence spectrum (Jasco 821-FP, Spectroscopic Co., Tokyo, Japan). The emission maximum was set at 500 nm and the excitation maximum at 398 nm. The assay was calibrated with authentic MG (Sigma M0377). Tumor necrosis factor_α _(TNF_α_) and interleukin-1_β _(IL-1_β_) were measured with antibody enzyme-linked immunosorbent assays (ELISAs) with a commercial antibody pair, recombinant standards, and a biotin-streptavdin-peroxidase detection system (Endogen, Rockford, IL, USA). All agents, samples, and working standards were prepared at room temperature according to the manufacturer's directions. The optical density was measured at 450/540 nm wavelengths by automated ELISA readers.

### Lung pathology

Lung tissue was fixed in 10% formaldehyde for 24 hr and then rinsed with tap water to remove formaldehyde. For light microscopic examination, lung tissue was dehydrated with graded alcohol and then embedded in paraffin at 60°C. A series of microsections (5 μm) was stained with hematoxylin and eosin. For quantification of lung injury score, we employed a modified grading method reported previously [6,24,25]. Various degree of lung injury score (LIS) were assessed as follows: degree 0, 1, 2, and 3 for no, mild, moderate and severe edema, respectively. For neutrophil and other cell infiltration, the scoring was similar to the evaluation of edema formation, degree 0-3 for no, mild, moderate and severe cellular infiltration. The histopathological assessment was performed in a blind fashion by several laboratory assistants. Each one gave a score for edema and cell infiltration from 0 to 3. The individual scores for edema and cell infiltration were added together to obtain a final score, ranging from 0-6.

### Detection of iNOS and eNOS mRNA in lung tissue

Reverse-transcriptase polymerase chain reaction (RT-PCR) was employed for a semiquantitative detection of iNOS and eNOS mRNA expression [26].

### Drugs

Phorbol myristate acetate (PMA) (Sigma Chemical, St Louis, MO, USA) was dissolved in dimethyl sulfoxide (DMSO; Sigma). Sivelestat (ONO-6818) was obtained from ONO company, Japan. It was dissolved in distilled water.

### Experimental protocol

A total of 40 isolated lungs was randomly divided into 4 groups. The vehicle group received DMSO 100 μg/g lung weight. In the FE group, corn oil 0.6 ml with distilled water 0.2 ml was added into the lung perfusate [5,6]. The FE+PMA group received corn oil micelles with PMA (4 μg/g lung weight) [27,28]. Sivelestat (10 μg/g lung weight) was coadministered with corn oil micelles in FE+SVT group. The dose was based on the dosage range from previous studies [29,30]. The isolated lungs were observed for 3 hr.

### Statistical analysis

All data were expressed as mean ± SEM. Comparisons within and among groups as well as data measurements over time in the same group were made using one-way analysis of variance with repeated measures, followed a *post hoc *comparison with Newman-Keuls test. Differences were considered to be statistically significant at *p *< 0.05.

## Results

### LW/BW ratio, LWG, exhaled NO and PCBAL

**Figure 1:**
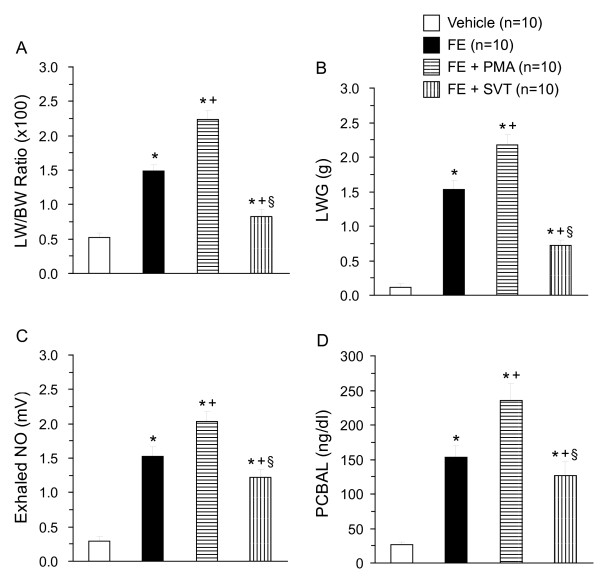
**Lung weight/body weight (LW/BW) ratio (A), LW gain (LWG) (B), exhaled NO (C) and PCBAL (D) in isolated perfused lung following fat embolism (FE), FE + phorbol myristate acetate (PMA) and FE + sivelestat (SVT)**. *p < 0.05 vs. vehicle; ^+^*p *< 0.05 FE + PMA vs. FE; and ^§^*p *< 0.05 FE + SVT vs. FE.

**Figure 2:**
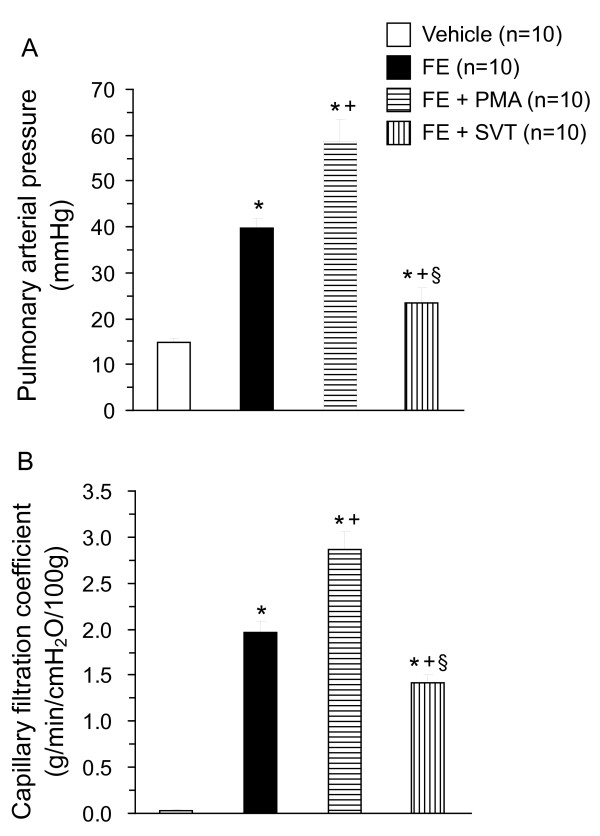
**Increases in pulmonary arterial pressure (A) and microvascular permeability (B) in isolated lung caused by FE**. Cotreatment with PMA enhanced the pulmonary hypertension and capillary filtration, while FE with SVT attenuated the changes in pulmonary arterial pressure and capillary permeability. *p < 0.05 vs. vehicle; ^+^*p *< 0.05 FE + PMA vs. FE; and ^§^*p *< 0.05 FE + SVT vs. FE.

**Figure 3:**
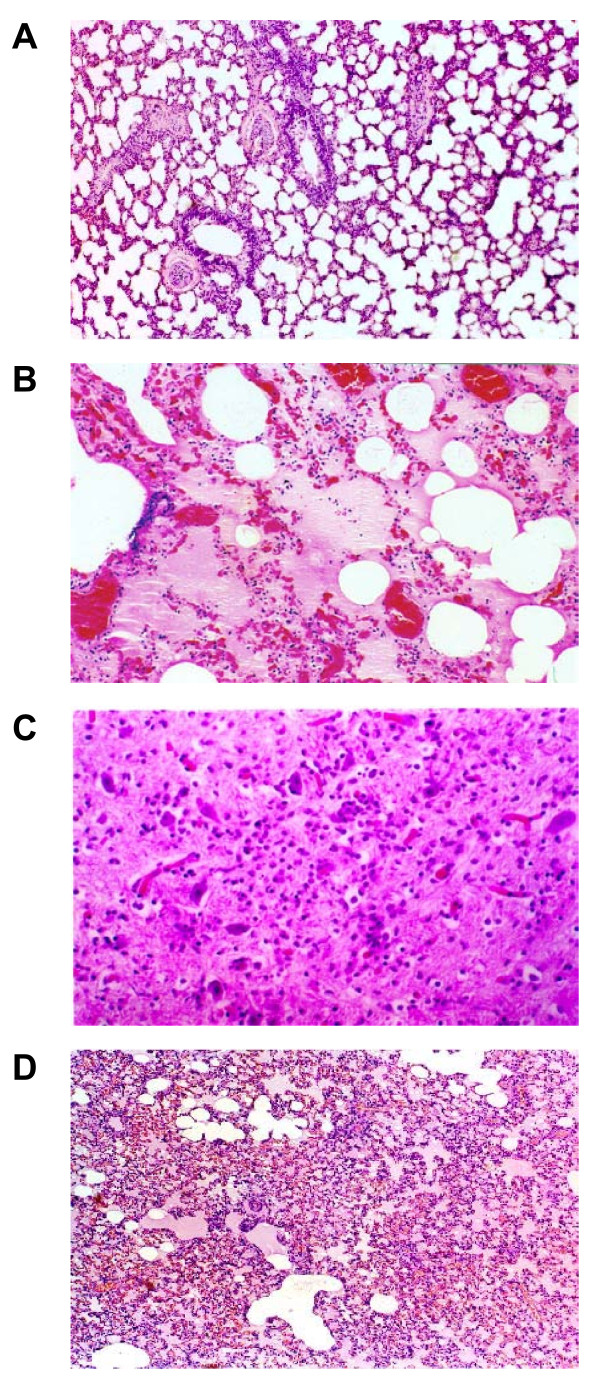
**Histopathology of the lung tissue in vehicle (A), FE (B), FE + PMA (C) and FE + SVT (D) groups**. FE caused severe alveolar edema and hemorrhage with inflammatory cell infiltration. Coadministration with PMA enhanced, but SVT diminished the lung pathology.

**Figure 4:**
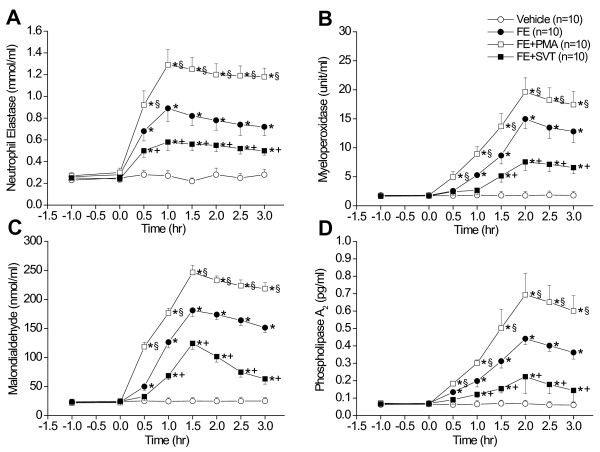
**Time course of changes in neutrophil elastase (A), myeloperoxidase (B), malondialdehyde (C) and phospholipase A_2 _(D) in lung perfusate of vehicle, FE, FE + PMA and FE + SVT groups**. *p < 0.05 vs. vehicle; ^+^*p *< 0.05 FE + PMA vs. FE; and ^§^*p *< 0.05 FE + SVT vs. FE.

**Figure 5:**
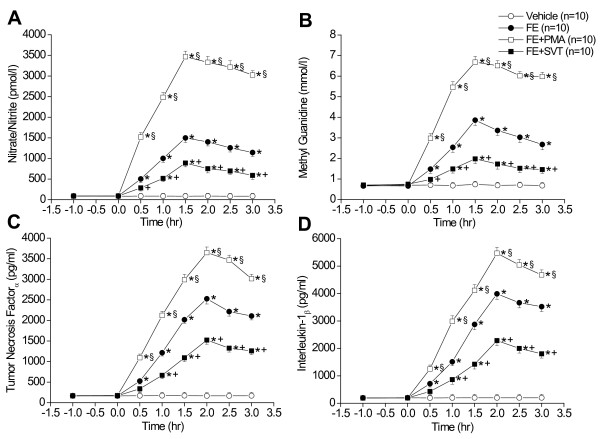
**Biochemical changes of nitrate/nitrite (A), methyl guanidine (B), tumor necrosis factor_α _(C) and interleukin-1_β _(D) in various groups**. FE significantly elevated these biochemical factors. Cotreatment with PMA exacerbated these changes, while FE with SVT mitigated the changes in these mediators. *p < 0.05 vs. vehicle; ^+^*p *< 0.05 FE + PMA vs. FE; and ^§^*p *< 0.05 FE + SVT vs. FE.

**Figure 6:**
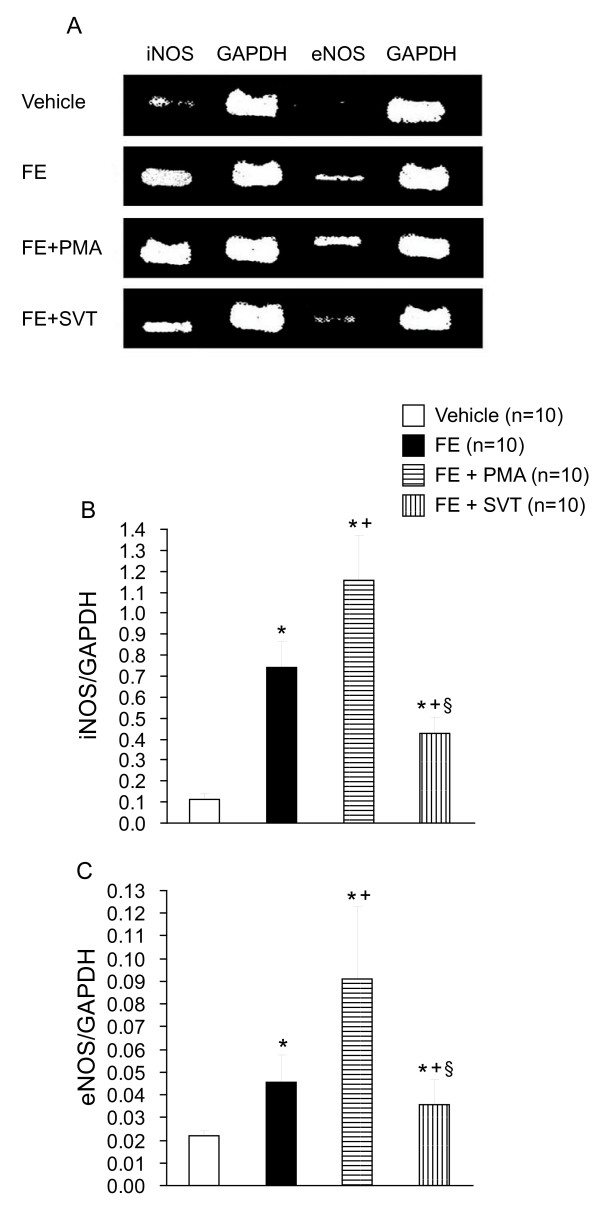
**Expression of inducible NO synthase (iNOS) and endothelial NO synthase (eNOS) mRNA by reverse transcriptase chain reaction in various groups (A)**. The iNOS/GAPDH and eNOS/GAPDH ratios (B) were increased by FE (*p < 0.05). Coadministration with PMA increased the iNOS and eNOS expression (^+^*p *< 0.05), while SVT attenuated the mRNA expression of iNOS and eNOS (^§^*p *< 0.05).

In the isolated perfused lungs, FE caused ALI as evidenced by the increases in LW/BW ratio, LWG, exhaled NO, and PCBAL (Figure 1). PAP and K_fc _were markedly elevated (Figure 2). Histopathological examination revealed severe lung edema and hemorrhage with inflammatory cell infiltration (Figure 3). The LIS was greatly larger than the vehicle control (Table 1). Biochemical determinations demonstrated significant increases in NE, MPO, MDA, PLA_2_, nitrate/nitrite, methyl guanidine, TNF_α_, and IL-1_β _(Figures 4 and 5). The time course demonstrated that NE reached it's peak at 1 hr after FE, MPO at 2 hr, MDA at 1.5 hr and PLA_2 _at 2 hr (Figure 4). Nitrate/nitrite peaked at 1.5 hr, methyl guanidine at 1.5 hr, tumor necrosis factor_α _and interleukin-1_β _at 2 hr (Figure 5). FE upregulated the iNOS expression significantly, while the eNOS was modestly increased (Figures 6A and 6B).

**Table 1:** The lung injury score

Vehicle	0.06 ± 0.02
FE	2.86 ± 0.12*
FE + PMA	4.93 ± 0.22*^+^
FE + SVT	1.42 ± 0.08*^+§^

Values are mean ± SEM (n = 10 for each group). *p < 0.05 vs. Vehicle; ^+^*p *< 0.05 FE + PMA and FE + SVT groups vs. FE group; ^§^*p *< 0.05 FE + SVT vs. FE + PMA. FE, fat embolism; PMA, phorbol myristate acetate; SVT, sivelestat.

Addition of PMA significantly exacerbated the ALI induced by FE. The values of LW/BW, LWG, exhaled NO, and PCBAL were elevated over those in the FE group (Figure 1). PMA also aggravated the FE-induced increases in pulmonary hypertension and capillary permeability (Figure 2). The lung pathology and LIS following FE were largely exacerbated by PMA (Figure 3 and Table 1). In addition, PMA aggravated the FE-induced increases in NE, MPO, MDA and PLA_2 _activities (Figure 4). It also increased the nitrate/nitrite, methyl guanidine, TNF_α _and IL-1_β _levels (Figure 5). This agent enhanced the iNOS expression significantly, while it elevated the eNOS mRNA modestly (Figure 6).

Administration of SVT significantly attenuated the FE-induced changes in lung parameters including LW/BW, LWG, exhaled NO, and PCBAL (Figure 1). It also reduced the PAP level and microvascular permeability (Figure 2). This neutrophil inhibitor abrogated the histopathological lesions of the lung and snignificantly reduced the LIS (Figure 3 and Table 1). The NE, MPO, MDA, PLA_2_, nitrate/nitrite, methyl guanidine, TNF_α _and IL-1_β _in lung perfusate were decreased by SVT (Figures 4 and 5). The NE inhibitor attenuated the FE-induced upregulation of iNOS significantly, whereas the eNOS expression modestly (Figure 6).

## Discussion

In the present study, we observed that fat embolism caused severe lung injury as evidenced by the changes in lung weight, exhaled NO, protein concentration in bronchoalveolar lavage fluid. In isolated lungs, FE caused severe lung injury associated with increases in pulmonary arterial pressure and microvascular permeability. The insult also resulted in increases in neutrophil elastase, myeloperoxidase, malondialdehyde and phospholipase A_2 _activities. In addition, FE elevated the nitrate/nitrite, methyl guanidine, tumor necrosis factor_α _and interleukin-1_β _in lung perfusate. These pulmonary and biochemical changed were enhanced by coadministration with PMA, while mitigated by SVT.

The pathogenesis of FE syndrome associated acute respiratory distress syndrome are complicated. Two clinical investigations from our laboratory have suggested that NO, phospholipase A_2_, free radicals and proinflammatory cytokines are involved in the chemical phase. The major source of NO is the alveolar macrophages [1,3]. Animal studies have revealed that N-acetylcysteine and NO inhibitors are able to abrogate the FE-induced changes [6,7]. The present study further provides evidence to indicate that NE inhibitor, sivelestat may be a therapeutic consideration for patients with FE syndrome.

Our observation of the time course of changes in mediators and proinflammatory cytokines may be valuable in the identification of the pathological subsequence of fat embolism. Neutrophil elastase may be released at the early chemical phase, followed by malondialdehyde, NO and hydroxyl radical (1.5 hr), then myeloperoxidase, phospholipase A_2_, tumor necrosis factor_α _and interleukin-1_β _(2 hr). To verify this contention and identify the consequence of inflammatory cascade following FE, further studies using transgenic animals and specific inhibitors are required.

The physical phase of fat embolism syndrome caused pulmonary hypertension and increased capillary permeability because of pulmonary microvascular obstruction [4,6]. We indeed observed two phases of increases in PAP and LWG, an initial sharp upstroke followed by a slow rise (not shown). The changes may reflect the early physical phase and the late chemical phase of pulmonary hypertension and increased K_fc_.

The increases in exhaled NO and nitrate/nitrite in lung perfusate, and NOS expression suggested that NO production was involved in the FE-induced ALI and associated changes. Our laboratory has reported that NO via the iNOS up-regulation contributed to the development of ARDS in children with enterovirus infection, patients with leptospirosis and scrub tybus and animal with hypercalcemia and endotoxemia [31-34]. In a murine model of sepsis, Razava et al. proposed up-regulation of iNOS in the lung caused neutrophil infiltration. The recruitment iNOS-positive neutrophil further aggravated the oxidative stress in the lung [35]. We have also reported that pretreatment of iNOS isoform inhibitors attenuated the inflammatory responses to endotoxin, fat embolism and PMA. On the other hand, NO donors enhanced the pathological and biochemical changes [7,27,36]. Accordingly, NO production through the iNOS system plays a detrimental role in endotoxemia, infections and fat embolism as well. The participation of eNOS in the FE-induced changes appears to be of less importance.

Neutrophil activation and recruitment leading to release of neutrophil elastase, myeloperoxidase and malondialdehyde and other mediators have been proposed to be a important step leading to an inflammatory cascade. Pulmonary hypertension and increased microvascular permeability ensued. These alterations finally resulted in damage of the alveolar capillary barriers, thus created severe lung injury [8,9,11]. In the present study, the neutrophil-derived mediators were significantly elevated following fat embolism. The facilitatory effects of phorbol myristate acetate, while the inhibitory effects of sivelestat on the pathophysiological, biochemical and molecular changes induced by fat embolism further support the importance of neutrophil activation and release of neutrophil-derived mediators on the acute lung injury induced by fat embolism. In addition, phospholipase A_2 _was increased in bronchoalveolar fluid and plasma of patients associated with fat embolism syndrome [2,3]. The ultimate role of phospholipase A_2 _in the FE-induced acute lung injury requires further investigation.

## Conclusions

Fat embolism caused acute lung injury, pulmonary hypertension, increased microvascular permeability and lung pathology. The challenge in isolated and perfused rat's lungs also significantly elevated the neutrophil elastatase, myeloperoxidase, malondialdehyde and phopholipase A_2 _in lung perfusate. In addition, fat embolism caused release of nitrate/nitrite, hydroxyl radical, tumor necrosis factor_α _and interleukin-1_β _from the lung. The time course revealed that neutrophil elastase was produced at the early chemical phase followed by other mediators. NO production through the iNOS exerts a detrimental effect on the lungs. Cotreatment with phorbol myristate acetate (a neutrophil elastase activator) exacerbated, while sivelestat (a neutrophil elastase inhibitor) attenuated the FE-induced changes. Neutrophil activation and neutrophil-derived mediators are involved in the FE syndrome associated with acute lung injury. Sivelestat may be recommended for clinical application in subjects with fat embolism syndrome.

## Competing interests

The authors declare that they have no competing interests.

## Authors' contributions

CCL and PHL performed the animal experiments. PHL also carried out pathological examination of the lung, and his assistants assessed the lung injury score. SJK was in charge of the data collection and analysis. HIC was the principal investigator to obtain the grant. He contributed to the study design, coordination, manuscript writing and correspondence. All authors read and approved the final manuscript.

## References

[B1] Hsu YH Kao SJ Lee RP Chen HI Acute pulmonary oedema: rare causes and possible mechanisms Clin Sci (Lond) 2003 104 259 264 10.1042/CS20020166 12605583

[B2] Karagiorga G Nakos G Galiatsou E Lekka ME Biochemical parameters of bronchoalveolar lavage fluid in fat embolism Intensive Care Med 2006 32 116 123 10.1007/s00134-005-2868-x 16322975

[B3] Kao SJ Yeh DY Chen HI Clinical and pathological features of fat embolism with acute respiratory distress syndrome Clin Sci (Lond) 2007 113 279 285 10.1042/CS20070011 17428199

[B4] White T Petrisor BA Bhandari M Prevention of fat embolism syndrome Injury 2006 37 S59 S67 16990062 10.1016/j.injury.2006.08.041

[B5] Liu DD Hsieh NK Chen HI Histopathological and biochemical changes following fat embolism with administration of corn oil micelles: a new animal model for fat embolism syndrome J Bone Joint Surg Br 2008 90 1517 1521 18978276 10.1302/0301-620X.90B11.20761

[B6] Liu DD Kao SJ Chen HI N-acetylcysteine attenuates acute lung injury induced by fat embolism Crit Care Med 2008 36 565 571 10.1097/01.CCM.0000299737.24338.5C 18216605

[B7] Kao SJ Chen HI Nitric oxide mediates acute lung injury caused by fat embolism in isolated rat's lungs J Trauma 2008 64 462 469 10.1097/TA.0b013e318058aa2e 18301216

[B8] Kinoshita M Ono S Mochizuki H Neutrophils mediate acute lung injury in rabbits: role of neutrophil elastase Eur Surg Res 2003 32 337 346 10.1159/000052215 11182617

[B9] Abraham E Neutrophils and acute lung injury Crit Care Med 2003 31 S195 S199 10.1097/01.CCM.0000057843.47705.E8 12682440

[B10] Lee WL Downey GP Leukocyte elastase: physiological functions and role in acute lung injury Am J Respir Crit Care Med 2001 164 896 904 11549552 10.1164/ajrccm.164.5.2103040

[B11] Kuraki T Ishibashi M Takayama M Shiraishi M Yoshida M A novel oral neutrophil elastase inhibitor (ONO-6818) inhibits human neutrophil elastase-induced emphysema in rats Am J Respir Crit Care Med 2002 166 496 500 10.1164/rccm.2103118 12186827

[B12] Murakami K Okajima K Uchiba M The prevention of lipopolysaccharide-induced pulmonary vascular injury by pretreatment with cepharanthine in rats Am J Respir Crit Care Med 2000 161 57 63 10619798 10.1164/ajrccm.161.1.9808142

[B13] Murakami K Cox RA Hawkins HK Schmalstieg FC McGuire RW Jodoin JM Traber LD Traber DL Cepharanthin, an alkaloid from Stephania cepharantha, inhibits increased pulmonary vascular permeability in an ovine model of sepsis Shock 2003 20 46 51 10.1097/01.shk.0000065768.72937.62 12813368

[B14] Inoue Y Seiyama A Tanaka H Ukai I Akimau P Nishino M Shimazu T Sugimoto H Protective effects of a selective neutrophil elastase inhibitor (sivelestat) on lipopolysaccharide-induced acute dysfunction of the pulmonary microcirculation Crit Care Med 2005 33 1814 1822 10.1097/01.CCM.0000172547.54086.AD 16096460

[B15] Hagiwara S Iwasaka H Togo K Noguchi T A neutrophil elastase inhibitor, sivelestat, reduces lung injury following endotoxin-induced shock in rats by inhibiting HMGB1 Inflammation 2008 31 227 234 10.1007/s10753-008-9069-z 18536984

[B16] Fujii M Miyagi Y Bessho R Nitta T Ochi M Shimizu K Effect of a neutrophil elastase inhibitor on acute lung injury after cardiopulmonary bypass Interact Cardiovasc Thorac Surg 2010 10 859 862 10.1510/icvts.2009.225243 20354035

[B17] Ando M Murai T Takahashi Y The effect of sivelestat sodium on post-cardiopulmonary bypass acute lung injury in a neonatal piglet model Interact Cardiovasc Thorac Surg 2008 7 785 788 10.1510/icvts.2008.177576 18596053

[B18] Kao SJ Wang D Yeh DY Hsu K Hsu YH Chen HI Static inflation attenuates ischemia/reperfusion injury in an isolated rat lung in situ Chest 2004 126 552 558 10.1378/chest.126.2.552 15302744

[B19] Stewart TE Valenza F Ribeiro SP Wener AD Volgyesi G Mullen JB Slutsky AS Increased nitric oxide in exhaled gas as an early marker of lung inflammation in a model of sepsis Am J Respir Crit Care Med 1995 151 713 718 7533602 10.1164/ajrccm.151.3.7533602

[B20] Su CF Liu DD Kao SJ Chen HI Nicotinamide abrogates acute lung injury caused by ischaemia/reperfusion Eur Respir J 2007 30 199 204 10.1183/09031936.00025107 17504797

[B21] Bioshop ML Janet LP Free Radicals in Clinical Chemistry 1996 Third Philadelphia, Lippincott

[B22] Chen HI Hsieh NK Kao SJ Su CF Protective effects of propofol on acute lung injury induced by oleic acid in conscious rats Crit Care Med 2008 36 1214 1221 10.1097/CCM.0b013e31816a0607 18379248

[B23] Kitsiouli EI Nakos G Lekka ME Differential determination of phospholipase A(2) and PAF-acetylhydrolase in biological fluids using fluorescent substrates J Lipid Res 1999 40 2346 2356 10588961

[B24] Chen HI Yeh DY Liou HL Kao SJ Insulin attenuates endotoxin-induced acute lung injury in conscious rats Crit Care Med 2006 34 758 764 10.1097/01.CCM.0000201902.37115.22 16505662

[B25] Kao SJ Liu DD Su CF Chen HI Niacinamide abrogates the organ dysfunction and acute lung injury caused by endotoxin J Cardiovasc Pharmacol 2007 50 333 342 10.1097/FJC.0b013e3180cbd18a 17878764

[B26] Lin NT Yang FL Lee RP Peng TC Chen HI Inducible nitric oxide synthase mediates cytokine release: the time course in conscious and septic rats Life Sci 2006 78 1038 1043 10.1016/j.lfs.2005.05.091 16181643

[B27] Yang YL Huang KL Liou HL Chen HI The involvement of nitric oxide, nitric oxide synthase, neutrophil elastase, myeloperoxidase and proinflammatory cytokines in the acute lung injury caused by phorbol myristate acetate J Biomed Sci 2008 15 499 507 10.1007/s11373-008-9238-y 18283562

[B28] Chu SJ Chang DM Wang D Hsu K Chiang CH Protective effect of lipophilic antioxidants on phorbol-induced acute lung injury in rats Crit Care Med 2001 29 819 824 10.1097/00003246-200104000-00028 11373476

[B29] Wang HG Shibamoto T Miyahara T Haniu H Tanaka S Fujimoto K Honda T Kubo K Koyama S Effect of ONO-5046, a specific neutrophil elastase inhibitor, on the phorbol myristate acetate-induced injury in isolated dog lung Exp Lung Res 1999 25 55 67 10.1080/019021499270420 10027079

[B30] Miyazaki Y Inoue T Kyi M Sawada M Miyake S Yoshizawa Y Effects of a neutrophil elastase inhibitor (ONO-5046) on acute pulmonary injury induced by tumor necrosis factor alpha (TNFalpha) and activated neutrophils in isolated perfused rabbit lungs Am J Respir Crit Care Med 1998 157 89 94 9445283 10.1164/ajrccm.157.1.9612021

[B31] Kao SJ Yang FL Hsu YH Chen HI Mechanism of fulminant pulmonary edema caused by enterovirus 71 Clin Infect Dis 2004 38 1784 1788 10.1086/421021 15227628 PMC7107954

[B32] Chen HI Kao SJ Hsu YH Pathophysiological mechanism of lung injury in patients with leptospirosis Pathology 2007 39 339 344 10.1080/00313020701329740 17558862

[B33] Chen HI Yeh DY Kao SJ The detrimental role of inducible nitric oxide synthase in the pulmonary edema caused by hypercalcemia in conscious rats and isolated lungs J Biomed Sci 2008 15 227 238 10.1007/s11373-007-9211-1 17906944

[B34] Hsu YH Chen HI Pulmonary pathology in patients associated with scrub typhus Pathology 2008 40 268 271 10.1080/00313020801911488 18428046

[B35] Razavi HM Wang le F Weicker S Rohan M Law C McCormack DG Mehta S Pulmonary neutrophil infiltration in murine sepsis: role of inducible nitric oxide synthase Am J Respir Crit Care Med 2004 170 227 233 10.1164/rccm.200306-846OC 15059787

[B36] Su CF Yang FL Chen HI Inhibition of inducible nitric oxide synthase attenuates acute endotoxin-induced lung injury in rats Clin Exp Pharmacol Physiol 2007 34 339 346 10.1111/j.1440-1681.2007.04553.x 17324147

